# Applications of machine learning in time-domain fluorescence lifetime imaging: a review

**DOI:** 10.1088/2050-6120/ad12f7

**Published:** 2024-02-08

**Authors:** Dorian Gouzou, Ali Taimori, Tarek Haloubi, Neil Finlayson, Qiang Wang, James R Hopgood, Marta Vallejo

**Affiliations:** 1 Dorian Gouzou and Marta Vallejo are with Institute of Signals, Sensors and Systems, School of Engineering and Physical Sciences, Heriot Watt University, Edinburgh, EH14 4AS, United Kingdom; 2 Tarek Haloubi, Ali Taimori, and James R. Hopgood are with Institute for Imaging, Data and Communication, School of Engineering, University of Edinburgh, Edinburgh, EH9 3FG, United Kingdom; 3 Neil Finlayson is with Institute for Integrated Micro and Nano Systems, School of Engineering, University ofEdinburgh, Edinburgh EH9 3FF, United Kingdom; 4 Qiang Wang is with Centre for Inflammation Research, University of Edinburgh, Edinburgh, EH16 4TJ, United Kingdom

**Keywords:** machine learning, fluorescence lifetime imaging, FLIm, deep learning, biomedical engineering

## Abstract

Many medical imaging modalities have benefited from recent advances in Machine Learning (ML), specifically in deep learning, such as neural networks. Computers can be trained to investigate and enhance medical imaging methods without using valuable human resources. In recent years, Fluorescence Lifetime Imaging (FLIm) has received increasing attention from the ML community. FLIm goes beyond conventional spectral imaging, providing additional lifetime information, and could lead to optical histopathology supporting real-time diagnostics. However, most current studies do not use the full potential of machine/deep learning models. As a developing image modality, FLIm data are not easily obtainable, which, coupled with an absence of standardisation, is pushing back the research to develop models which could advance automated diagnosis and help promote FLIm. In this paper, we describe recent developments that improve FLIm image quality, specifically time-domain systems, and we summarise sensing, signal-to-noise analysis and the advances in registration and low-level tracking. We review the two main applications of ML for FLIm: lifetime estimation and image analysis through classification and segmentation. We suggest a course of action to improve the quality of ML studies applied to FLIm. Our final goal is to promote FLIm and attract more ML practitioners to explore the potential of lifetime imaging.

## 1. Introduction

### 1.1. Fluorescent lifetime imaging

Fluorescence is a form of light emission produced from the shift to excited states happening subsequently to the absorption of photon energy from an external light source. The phenomenon exhibits emission and excitation spectra characterised by different metrics such as intensity and lifetime. Lifetime is the average time between excitation and return to the ground state. Fluorescence Lifetime Imaging (FLIm) is a valuable bio-imaging approach for investigating the behaviour and interaction of very small-scale objects, such as molecular and cellular microorganisms. In this technique, a sample is first excited by the focused light of a laser. The electrons inside the sample get stimulated and move from a calm, ground state to an excited state. Then, the fluorophore molecules fluoresce. The fluorophore photons are captured by a detector and the temporal responses are captured across different optical wavelengths and used to create the FLIm images by characterising the fluorescence lifetime from their decay profile [1].

FLIm devices have important, diverse applications, including diagnosis and prognosis, where it has shown great success in identifying tumours and delimiting them, such as oral cancer [2, 3] or breast tumour [4, 5], a task for which FLIm is a candidate, among other technologies, to replace or complement histopathologic analysis. More broadly, it is also utilised for drug monitoring, obtaining penetration of micro- and nano-particles information, visualising topical drug uptake *in vivo* [6–8], disease treatment [9, 10] or other biomedical tasks such as sperm assessment [11].

As opposed to fluorescence spectroscopy or other imaging techniques, FLIm does not produce an image directly. For each ‘pixel’, the lifetime is estimated from the characterised decay curve, often for multiple spectral channels, as discussed in section 2.6. In FLIm, lifetime information obtained at each pixel complements per-pixel measurements of fluorescence intensity, enabling differentiation of image regions which cannot be obtained from intensities. FLIm sensing can be carried out in both time [12] and frequency domains [13], which differ in terms of hardware and software aspects. Time-domain FLIm systems use pulsed lasers for excitation and exploit transient temporal responses to estimate the fluorescence lifetime with the aim of fast imaging, whereas frequency-domain FLIm utilises sinusoidal excitation and measures the phase shift of the wave [14] to determine the lifetime indirectly. This review narrows its focus more on developed time-domain systems.

### 1.2. Artificial intelligence and machine learning

Artificial Intelligence (AI) refers to the ensemble of methods aiming to make computers solve problems. It is divided into different categories such as natural language processing, evolutionary computation or Machine Learning (ML). ML encompasses a set of techniques designed to make a machine ‘learn’ from data how to perform one or multiple given tasks [15]. Deep Learning (DL) is a subset of ML which makes use of a large amount of data to train Artificial Neural Network (ANN) with multiple layers, alternating linear computation and non-linear activation function, the adjective deep denoting the difference with ANN used in ML which do not have intermediary layers.

ML and DL are very powerful tools for data analysis [16], and promoted the progress of many complex tasks such as text processing and translation [17, 18] or picture captioning [19] through multimodel learning. Another successful area of applicability is medical and biomedical imaging. Examples of such use are Magnetic Resonance Imaging (MRI) [20], Electroencephalography (EEG) [21], Optical Coherence Tomography (OCT) and OCT-Angiography [22, 23], biomedical optics modalities [24] or generally for task such as segmentation [25].

ML includes a wide variety of algorithms, also called ‘models’, trained in a standardised way. The most common and easiest training type is supervised learning, which includes two or three stages: (1) training, in which the model is exposed to the data to learn its patterns; (2) testing, when the trained model is exposed to new, unseen data and is evaluated on how well it performs the assigned task; and (3) validation, sometimes not performed, which is used to select the best model, either among different ML modalities or by selecting the best hyper-parameters for a specific model. The evaluation methods are problem-dependent. On the other side, in unsupervised learning, the models make use of unlabelled data to make their own representation of them, either to group them by similarity or to mimic them [26].

**Figure 1.**
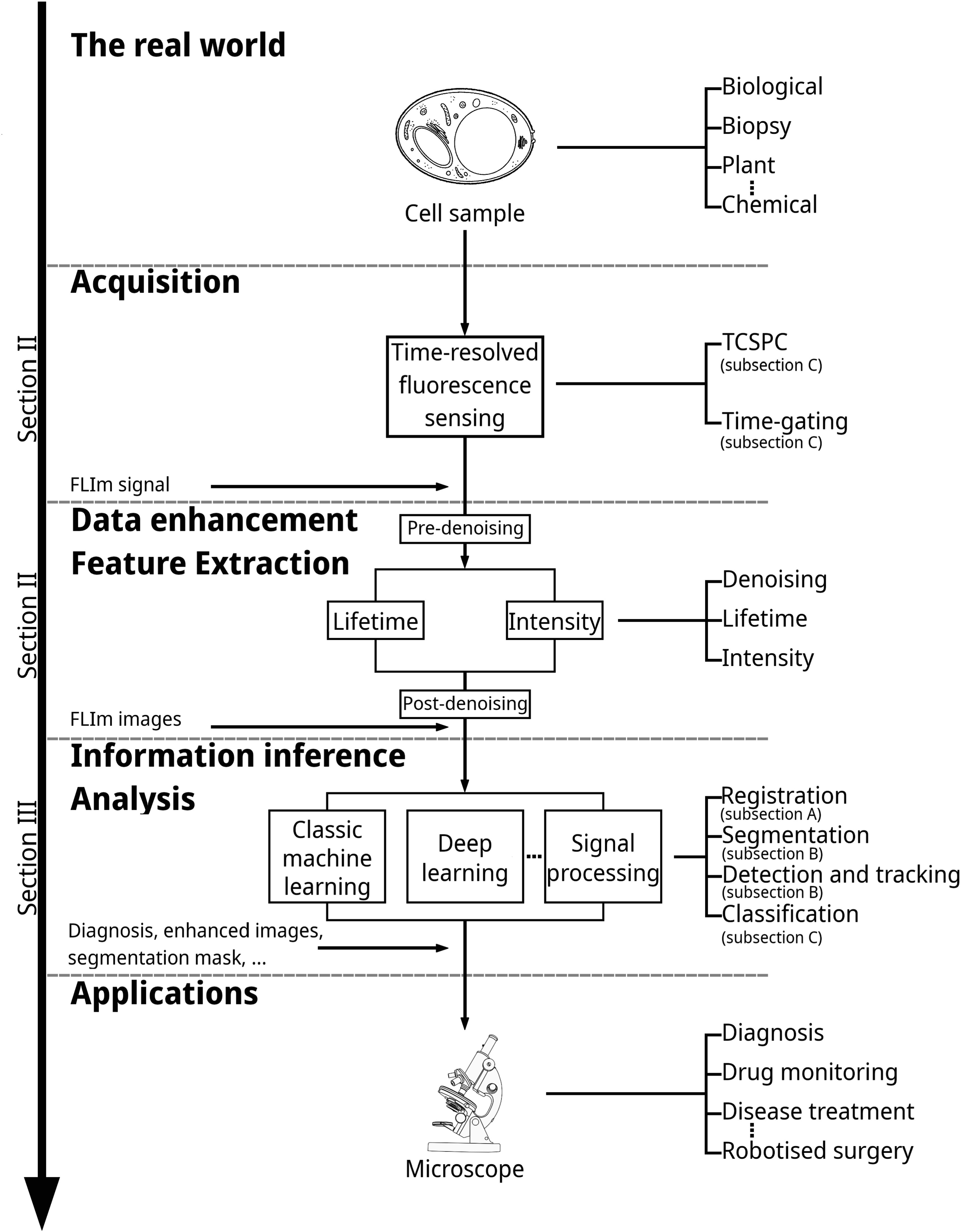
The structure of this paper follows an illustration of the FLIm data acquisition and processing general pipeline.

**Figure 2.**
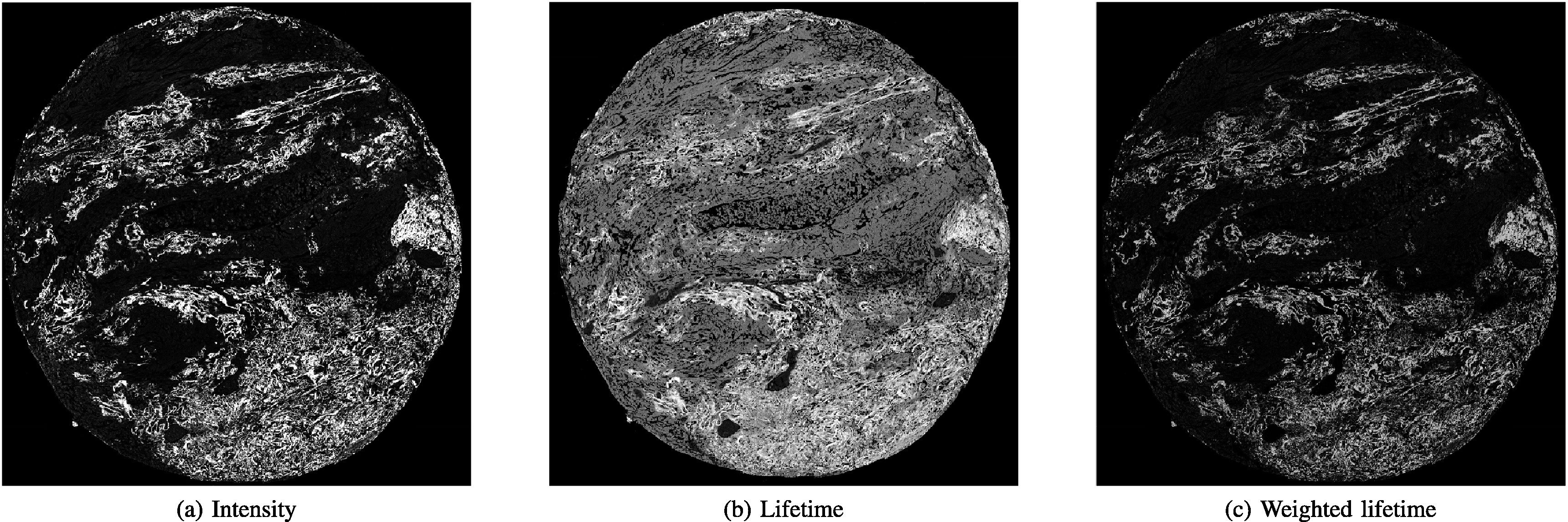
Lungs tissue FLIm scan, obtained on cancerous sample with Leica SP8 system. (a) Intensity, (b) lifetime (c) and weighted lifetime with intensity images of biological tissue.

Now, it is widely considered that ML-based techniques will be key to addressing many medical and biomedical challenges and improving diagnostic techniques. Specifically, FLIm benefits from progress in ML, whether by reducing the computation load needed to characterise lifetime or by automated processing of FLIm images which are not humanly understandable today. Our aim is to address this important subject and review researches that fall into this category in a structured manner as schematised in figure 1. We separated signal processing and raw data studies from image analysis, according to the evolution of the data through the multiple processing stages. Figure 2 also gives a general view to readership how output images of a FLIm system on a sample look like.

### 1.3. The purpose and synergy of this paper

In the literature of FLIm, several valuable review papers explain the underlying physical processes and applications of FLIm exist [9, 13, 27–32]. Recently, a few researches mentioned some applications of ML to FLIm [24, 33–35]. We aim to complement those studies by providing an up-to-date, detailed and structured review exclusively focused on FLIm.

This review highlights the importance of ML in medical research, summarises the current state of the ML applications in FLIm and focuses on recent advances made in FLIm since those reported by Marcu [36] and Becker [28] a decade ago. In this paper, we will review applications of regression and classification problems for lifetime estimation and mostly disease classification, including cancer. Regression uses criteria such as bias and variance for evaluating estimation efficiency, whereas classification employs metrics like sensitivity and accuracy to evaluate how well a model classifies the unknown element or differentiates the different classes under investigation.

### 1.4. Paper organisation

The rest of this paper is organised as indicated in figure 1. Section 2 addresses FLIm data acquisition and enhancement, as well as the FLIm feature extraction and ongoing studies of ML-based model for lifetime estimation. It presents FLIm to beginners in this field and introduces important resources. Section 3 reviews research in FLIm images analysis. It explains to ML researchers what the different ML tasks are in FLIm, summarises current methodologies and provides advice for more advanced ML studies. Finally, section 4 concludes the paper by remarking on key challenges and opportunities in the field.

## 2. Fluorescence lifetime image acquisition

### 2.1. An early history of FLIm related research

The foundations and applications of biomedical photonics, fluorescence spectroscopy, and Time-Correlated Single Photon Counting (TCSPC) supporting time-resolved fluorescence are described in seminal texts by Chance *et al* [37, 38], Lakowicz *et al* [1, 39].

Fluorescence spectroscopy and imaging often employ targeted fluorescent labels or probes called exogenous fluorophores. Another approach is the investigation of auto-fluorescence tissue properties through endogenous fluorophores. For example, the coenzymes Nicotinamide Adenine Dinucleotide (NADH) and Flavin Adenine Dinucleotide (FAD) enable label-free detection of metabolic changes and interpretation of cellular metabolic states [40], and are extensively used in FLIm images analysis with ML. FLIm has also shown that it is able to detect, at pixel resolution, abnormal cellular metabolism through the level of activation of oxidative phosphorylation measured as an increase in the fraction of protein-bound Nicotinamide Adenine Dinucleotide (NAD), yielding important information about cell responses in metabolic diseases and cancer [41, 42].

In 1962, Chance *et al* reported a study where fluorescence emission was used to study the localized metabolic response of tissue to variation in oxygen concentration, interpreting the cellular metabolic state in terms of the relative amount of oxidised and reduced form of NADH [37]. However, the emission spectra of many tissue fluorophores overlap. FLIm is a technique which enables such overlapping fluorophores to be distinguished by their lifetime. Examples of such techniques are given by Lakowicz *et al* [39], who used the FLIm technique to exploit the high information content of Time-Resolved Fluorescence (TRF) in a 1992 study of free and protein-bound NADH metabolites. Subsequently, Skala *et al* [43] investigated early stages of cancer development through a combination of cellular redox ratio, NADH and FAD lifetime, and subcellular morphology *in vivo* imaging [44].

Naturally, early devices of FLIm employed traditional approaches of computing. However, with the current progress in computational power, a new generation of them is equipped with AI and ML.

### 2.2. Foundation

Confocal beam scanning is a microscopy technique first patented by Minsky [45] and later developed by Petrán *et al* [46], Davidovits and Egger [47] and Sheppard and Choudhury [48]. The technique uses object and image plane pinholes to reject unwanted scattered background light, building an image pixel-by-pixel with a raster scan typically using scanning galvanometer mirrors and is now used widely in FLIm applications. TCSPC-based FLIm utilising a laser confocal scanning microscope was first described by Bugiel *et al* [49]. Wide-field FLIm in contrast is a technique where time-resolved signals in all pixels are acquired in parallel [50] yielding greater frame rates at the cost of increased background light levels.

A key goal for clinical research applications is *in vivo* FLIm, where video rates of frame acquisition are desirable to avoid motion artefacts. In 2005, Munro *et al* [51] demonstrated high-speed video-rate FLIm through a flexible endoscope, pointing the way to clinical applications of such techniques. Wide-field FLIm was exploited to achieve high acquisition rates based on gated optical image intensifier technology. Elson *et al* [52] investigated forward- and side-viewing FLIm fibre bundles comprised of 10,000 coherent fibres.

For fluorophores requiring excitation in the UV range, it is necessary to utilise two-photon excitation [53]. NADH for example absorbs light in the 335–350 nm range, with an emission peak around 440–470 nm [54].

TCSPC originated in 1960 with Bollinger and Thomas [55]. Multidimensional TCSPC signals as a function of wavelength, polarisation and other quantities became possible with multiple detector channels in 1993, and TCSPC acquisition times became 100 times faster. TCSPC became combined with microscopy [56]. The last two decades have seen major advances in detector technologies. Single-Photon Avalanche Diode (SPAD) arrays emerged in standard Complementary metal-oxide-semiconductor (CMOS) technology in 2003 [57] and time-resolved SPAD detection is now one of the most important and rapidly advancing technologies enabling advances in FLIm [52].

Comprehensive reviews of fluorescent lifetime techniques in medical applications with an emphasis on translational research potential supporting *in vivo* characterization and diagnosis of disease are provided by Marcu [36] and Alfonso-Garcia *et al* [58]. Other reviews include Suhling *et al* [29] and Liu *et al* [27]. Marcu provides a detailed overview of time-resolved fluorescence techniques in biomedical diagnostics and the development of Time-Resolved Fluorescence Spectroscopy (TRFS) and FLIm instrumentation and methodologies. A key concern is evaluating whether intrinsic label-free endogenous fluorescence signals from biological tissues provide sufficient contrast for the diagnosis of cancers of the gastrointestinal tract, lung, head and neck, brain, skin and eye disease, and cardiovascular disease. Marcu lists 63 *in vivo* autofluorescence studies in humans, classified by organ investigated, method, excitation and emission ranges, power levels and numbers of patients.

Other significant reviews include those on FLIm, reviewed by Berezin and Achilefu [9], König [59], Datta *et al* [30] and Liu *et al* [27]; on wide-field FLIm Hirvonen and Suhling [50]; on the role of optical fibre technologies in clinical imaging of lung cancer Fernandes *et al* [40]; on fluorescence-guided surgery Stewart and Birch [60]; on CMOS SPAD time-resolved detection technologies, reviewed by Bruschini *et al* [61], Gyongy *et al* [62]); and, of most relevance to this review, on ML analysis of biomedical images Cao *et al*[63].

### 2.3. Fluorescence lifetime imaging sensors

SPAD arrays, developed in standard CMOS solid-state technology since 2003 [57], support a host of biophotonics applications [61]. SPAD detectors offer single photon spectroscopy and imaging capabilities with unparalleled photon counting and time-resolved performance when compared to CCDs/-sCMOS imagers. Among the advantages of CMOS SPAD arrays [61, 62] are highly parallel single-photon counting and photon timing, integration with standard digital blocks for data acquisition and/or processing, absence of read-out noise, very high frame rate binary implementations for real-time capture of fast transient phenomena and time-gating/binning to support Raman spectral acquisition in the presence of strong fluorescence [64, 65]. A key advantage of CMOS integration is that smart sensor architectures become possible, which include on-chip, and indeed in-pixel, timestamping, histogramming and signal processing functionality.

SPAD devices leverage decades of investment in scalable, high-yield CMOS technologies and are exhibiting rapid improvements in key parameters such as Photon Detection Probability (PDP) and fill factor, dark count rates (DCR), spatial resolution (quarter megapixel, with megapixel arrays on the horizon), shrinkage of pixel size to well below 10 *μ* m and excellent timing accuracy (typically 50-100 ps, with the best SPADs in the 20-30 ps range).

**Figure 3.**
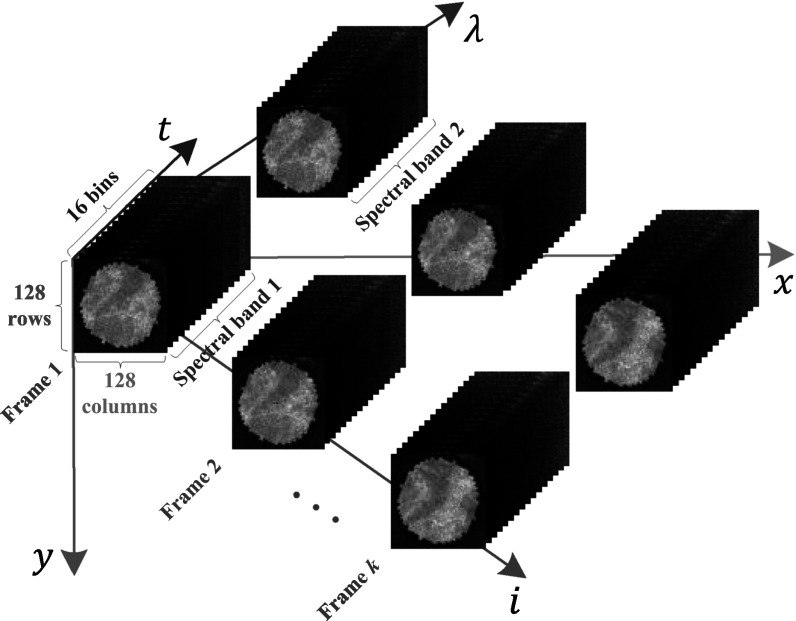
Tensor data formatting in a representative two-spectral FLIm system. The dimensions along (*x*, *y*), *t*, *λ*, and *i* represent spatial coordinate, time bin, wavelength, and video-frame sequence index, respectively.

A CMOS SPAD line sensor for FLIm application was described by Erdogan *et al* [66]. This sensor supports 512 spectral channels, single photon counting and on-chip per-pixel TCSPC histogramming with up to 32 time bins. The sensor was subsequently used for FLIm [12] and combined fluorescence lifetime/Raman spectroscopy [65]. To visualise the hardware output format, figure 3 shows the arrangement of raw data in a FLIm system with the capability of generating multi-spectral video sequences. The data representation is considered as a 5D tensor arrangement consisting of a matrix of cubes as (*x*, *y*; *t*; *λ*; *i*). The dimensions along (*x*, *y*), *t*, *λ* and *i* denote spatial coordinate, bin time, wavelength and frame sequence index, respectively. In *ex vivo* microscopy applications, the sensor generates very large *x* × *y* × *t* × *λ* = 256 × 256 × 16 × 512 cube per frame, which facilitate detailed investigation of the dispersion of fluorescence lifetime in tissue and Raman and fluorescence spectroscopic correlations. Biophotonics applications include (endoscopic) FLIm, (multi-beam multiphoton) FLIm-Förster resonance energy transfer (FRET), SPIM-FCS, super-resolution microscopy, time-resolved Raman NIROT and positron emission tomography.

ML associated with SPADs is receiving increased attention [64, 67]. In [64], a fluorescence-suppressed Raman spectrometer utilising 16256 time-resolved CMOS SPADs was employed with K-means, an unsupervised clustering model to obtain high-quality images of human teeth. K-means is a low-complexity algorithm, easy to implement but which provides good results. It was used to identify regions in the images without clinical labelling. While it is effective and perfect for proof of concept work as this one, recent approaches in DL such as unsupervised Convolutional Neural Network (CNN) for segmentation, promise improvements in image and data quality [68].

### 2.4. Spatio-temporal-spectral sensing and early-stage processing

Similar techniques can be adopted in both frequency- and time-domain for measuring the physical parameter of fluorescence lifetime. E.g, the Fourier space approach of phasors [69, 70]. Focusing on time-domain systems, two basic forms of time-determined fluorescence (aka TRF) can be described, namely time-gating [71] and TCSPC [72]. From a data analysis perspective, FLIm systems designed by the above techniques mainly differ in terms of temporal resolution and video frame rate [14]. Generally, the temporal resolution (or the time bin resolution) in TCSPC is higher than time-gating [73]. However, the frame rate of the time-gating technique is typically higher than that of TCSPC. Other key attributes of TCSPC are low-noise and wide bandwidth characteristics [72], which make it utilisable in a broad range of applications [65].

In FLIm, a point of a target specimen is excited by a focused laser, yielding emission spectra extending typically within visible light range. The first emitted photon from an excited location that can reach the sensing equipment is detected, and then its arrival time is measured. Repeating the process for a given measurement period leads to a set of timestamps in each spectral channel. Before determining the lifetime, the observations must be pre-processed by histogramming of the timestamps. In this process, a number of bins or time gates are available with a given temporal resolution. Subsequentially, timestamps falling into any individual bin interval are counted. Finally, an estimation of the distribution of arrived photons (called histogram of photon counts) is calculated. The whole step may be done on-chip in the detection unit [66] or on a destination computer in order to generate a histogram from photon counting. A scanning for all points of the sample by the above procedure provides spatial information.

Both time-gating and TCSPC techniques can output the histogram of photon counts as a function representing the fluorescence decay curve. This temporal sensing may be only a time series from a single point on a sample, e.g., a resultant reactional response obtained from a chemical sample in spectroscopy applications [65], or pixel-by-pixel imaging of a biological tissue section [12]. Regardless of the details behind the electronics of the FLIm system, what is important for machine learners and signal processing experts is the formatting of its output data, where algorithms import the raw data for diverse purposes ranging from signal processing tasks to visual feature extraction.

### 2.5. Lifetime benchmark dataset

One important issue in FLIm is the availability of experimental datasets from known reference fluorophores. Benchmark datasets facilitate measuring the efficiency of a lifetime estimator in terms of mean, as a measure of accuracy or bias, standard deviation, as a measure of precision, and Signal-to-Noise Ratio (SNR), as a measure of signal strength. Chemical dyes or generally any samples with a plain property on image plane [74] can be appropriate options for a lifetime benchmark dataset. Fluorescein, Rhodamine B, Coumarin 6, Lucifer Yellow and 9-Cyanoanthracene are prominent fluorescent dyes [75, 76]. Partial information about the behaviour of fluorophores is already available, which helps in calibration. However, it should be noted that they are not sufficient for training (deep) supervised approaches, where multifluorophore data with a range of lifetime values are required. As will be discussed in the next section, learning-based methods often rely on synthetic data to solve the problem of lifetime estimation [77, 78].

### 2.6. Lifetime estimation

**Figure 4.**
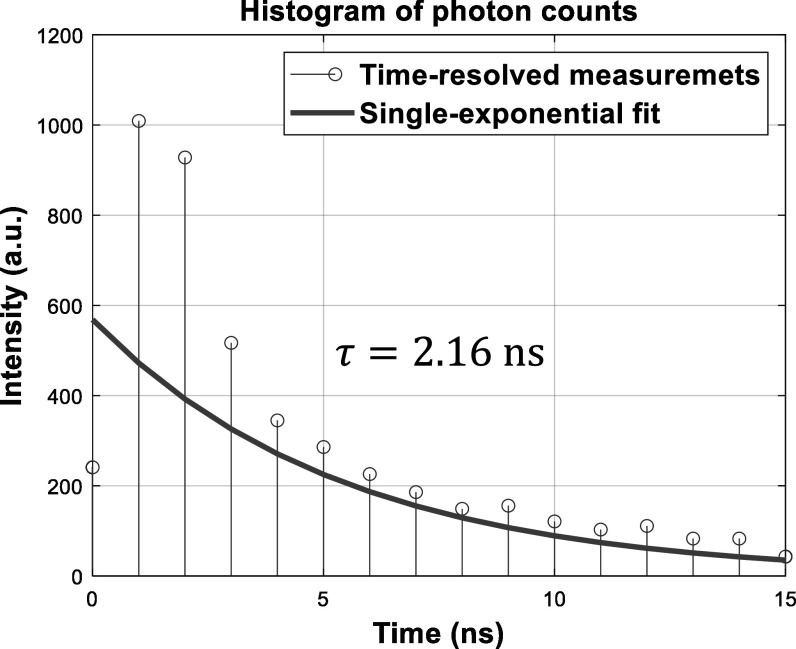
Illustration of the fluorescence lifetime, *τ*, estimation from real data.

The histogram of photon counts is recorded for each pixel of a FLIm system. The fluorescence lifetime, as an important molecular bio-marker representing the decay rate of the histogram function, is estimated from the photon measurements. Figure 4 illustrates the process of lifetime estimation of a pixel. Estimated lifetimes over all pixels discriminate objects and regions of interest in molecular environments, where they cannot otherwise be easily distinguished on the basis of intensity of emitted photons. Based on the behaviour of fluorophores, the shape of the function may be mathematically modelled by a single- [74], double- [79], tri- [80], or generally multi-exponential decay curve. E.g., the function:

\begin{eqnarray*}x[n]=A\left[{\underbrace{\alpha }}_{\triangleq {\alpha }_{1}}{e}^{\tfrac{-{\mathrm{\Delta }}\cdot n}{{\tau }_{1}}}+{\underbrace{(1-\alpha )}}_{\triangleq {\alpha }_{2}}{e}^{\tfrac{-{\mathrm{\Delta }}\cdot n}{{\tau }_{2}}}\right],\forall n=0,1,...,N-1,\end{eqnarray*}

represents a double- or bi-exponential model, in which $A\in {{\mathbb{R}}}^{+}$, 0 < *α* < 1, ${\tau }_{1}\in {{\mathbb{R}}}^{+}$ and ${\tau }_{2}\in {{\mathbb{R}}}^{+}$ represent initial amplitude, pre-exponential factor, short and long lifetimes, respectively. The parameters Δ and *N* denote the time bin width and the number of histogram bins, respectively. The symbol ${{\mathbb{R}}}^{+}$ represents the set of all positive real numbers. Setting *α* = 1 in equation (1) reduces it to a single- or mono-exponential function. In practice, the measurements are affected by noise from different sources as well as deformation in the histogram due to the convolution of a given model and the Instrument Response Function (IRF) [74]:

\begin{eqnarray*}I[n]=\lfloor x[n]* h[n]+\eta [n]\rceil ,\end{eqnarray*}

where the round operator of ⌊ · ⌉ delivers quantised photon counts, the symbol * is convolution operator, and functions *I*[*n*], *h*[*n*] and *η*[*n*] signify fluorescence intensity, IRF and additive noise term, respectively. At a given wavelength *λ*, the intensity in terms of photons per histogram in sensing or photons per pixel (p/p) in imaging can simply be measured by summing photons across all bins as:

\begin{eqnarray*}{I}_{\lambda }\triangleq \displaystyle \sum _{n=0}^{N-1}I[n].\end{eqnarray*}

These perturbations to the underlying signal make the problem of lifetime estimation a complicated task, especially in low photon count regimes with the intensity *I*_*λ*_ around 100 to 200 photons [78]. For the model presented in (1), it can be shown that the expected (mean) lifetime is:

\begin{eqnarray*}{\tau }_{\mathrm{mean}}=\displaystyle \frac{\alpha {\tau }_{1}^{2}+(1-\alpha ){\tau }_{2}^{2}}{\alpha {\tau }_{1}+(1-\alpha ){\tau }_{2}}.\end{eqnarray*}

A considerable number of researchers measures a special weighted averaging formula as *τ*_avg_ ≜ *α* *τ*_1_ + (1 − *α*)*τ*_2_ instead, which has been already flagged to be incorrect in Lakowicz’s book [1]. The task of determining (4) can be translated back into a parameter estimation problem in regression or time series analysis. Different approaches have been proposed for lifetime estimation in the FLIm literature, including those enumerated by a recent review of [33] published in 2022. In this section, we provide a complementary critical review focusing more on detailed technical characteristics of developed AI- and ML-based approaches.

We divide the methods described in the literature into two broad categories of unsupervised and supervised methods. It should be noted that in information analysis/inference tasks of FLIm, any lifetime estimator from the above-mentioned types can be potentially employed as will be seen in section 3.3. Supervised lifetime estimators exploit learning data for pre-training, whereas unsupervised approaches do not require such data. Unsupervised methods can be grouped into two subcategories based on their nature, namely generic non parametric [81, 82] and statistical signal processing techniques [76, 83–91]. Generic processing approaches such as Rapid Lifetime Determination (RLD) [81] and RLD with overlapping windows [82] seek closed-form mathematical solutions to the fluorescence lifetime, whereas statistical ones such as Least Squares (LS), Maximum Likelihood Estimation (MLE) and Centre of Mass Method (CMM) are constructed upon estimation theory assuming a parametric model [92]. In this regard, optimised statistical estimators in LS [83, 84] and MLE [86–88] families are known as gold standard techniques of lifetime estimation. In Laguerre expansion-based lifetime estimation techniques [76, 91], the histogram of photon counts in each pixel is modelled by a linear combination of Laguerre basis functions. In fact, each of the two single-frequency exponential components in a bi-exponential model can be represented as Laguerre bases. Then, the problem of decay rate determination in the bi-exponential function is converted to statistically estimating corresponding Laguerre parameters. Statistical approaches in [89, 90] tried to jointly estimate the IRF of *h*[*n*] and fluorescence lifetime components from time-resolved data using iterative methods of extended Kalman filter and expectation maximisation, respectively. Unsupervised approaches have the potential to be used in the hardware realisation of FLIm systems [33], so long as they support real-time processing constraints such as existing approaches in RLD [74, 81, 82] or center of mass estimation [85]. However, a number of researchers have recently shifted the custom paradigms of lifetime estimation to learning-based mechanisms to specifically profit from the past decade of progress in DL methods, leading to the second supervised category [77–80, 93–96]. Supervised approaches target the function approximation capability of artificial Neural Network (NN) available in both subcategories of shallow and deep networks for fluorescence lifetime estimation. One of the outstanding deep learning models widely employed for estimating the lifetime [79, 80, 95, 96] is CNN. The architecture of a CNN consists of two main block of: (1) convolutional layers, as a representation learner; and, (2) Fully Connected (FC) decision layers, as a classifier/regressor. The representation block learns a set of dimensionality-reduced, influential features from raw input data in an automatic manner in connection with the classification/regression block. The classifier/regressor trains networks’ weights based on desired parameters. Table 1 tabulates important characteristics of AI- and ML-based lifetime estimators from a set of selected references. Specifically, the table describes each approach in terms of utilised architecture, input/output data format, training/test dataset splitting, decay model, noise model, learning/test speed and performance metrics.

**Table 1.** A summary overview of important characteristics of developed AI- and ML-based lifetime estimators.

Methodology	Architecture	Data format		Dataset		Decay	Noise	Speed metric		Performance
		Input	Output	Training	Test		model	Learning	Run-time^a^	
MLP [93]	2 hidden layers	57-bin histograms	2 pre-exponential coefficients	.21k histograms	Synthesised histograms	Bi-exp	Poisson	4 h	180 ×	Success Rate =99.93%
			2 lifetime terms		Real images					

CNN [79]	Pedestal branch for extracting temporal correlations	3D tensor (*x*, *y*; *t*)	Three 2D maps including images of amplitude	8k random data^b^	2k validation data	Mono-exp	Poisson	Hours	∼94*k* × ^c^	MAE =.083 ns (LPC)
	3 subsequent parallel branches for spatial filtering and regressing		2 lifetime components		Real images	Bi-exp				

FC NN [77]	3 hidden layers with (100, 50, 25) nodes	.2 bbin decays^d^	Lifetime	2*M* histograms	1M synthesised histograms	Mono-exp	Poisson + Gauss	38 min	1k×	MSE=.0053*ns* ^2^
	ReLU activation function				Actual experimental data					

1D CNN [80]	A main branch including 2 residual networks	*N*-tuple 256 bins	3, *N* × 1 arrays	32k decays	8k decay curves	Bi-exp^e^	Poisson	.5 h	300×	*F*-val =(35.5, 5, 79.8)^f^
	3 subsequent parallel CNNs									

GAN [78]	G: 2 convolutional+pooling layers and 3 FC layers^g^	G: 256 decay points, 256 IRF points	G: 256 outputs	.99k samples	Synthesised samples	Bi-exp	Not reported	G + D = 6.1 h	258 ×	MSE=.21 ns^2^ (50 p/p)
	D: 3 FC hidden layers	D: 256 decay points	D: 1 neuron		2 experimental labeled sets of 256 × 256 or 512 × 512 images with 256 time bins					
	E: 2 FC NNs + a concatenation layer+a hidden layer FC NN	E: 256 decay points, 256 IRF points	E: 3 neurons including amplitude and 2 lifetime components							
Robust RLD [74]	Adaptive multi-bin parallel lifetime estimators	Photons’ histogram	Recovered decay	Unsupervised	Synthesised histograms	Mono-exp	Poisson + Gauss	0	∼63 ×	Acc. ∼28% + (LPC)
	A game-theoretic ‘amplitude-lifetime’ fuser				Real 128 × 128 FLIm images with 16 bins					

^a^ This metric shows the amount of run-time improvement than the standard LS.

^b^ The authors inserted a histogram of photon counts into the well-known MNIST character recognition database.

^c^ This approach is also about 30 times faster than SPCImage commercial software.

^d^ The authors also trained and tested another neural network for 30-bin decay curves. Decays in this research are represented as normalised forms.

^e^ For tri-exponential decay model, the research has also been extended to another specific architecture.

^f^ The triple in *F*-val is (*τ*_1_, *τ*_2_, *α*).

^g^ Here, the acronyms of G, D and E stand for Generator, Discriminator and Estimator models, respectively.

The seminal research of Wu *et al* [93] developed in 2016 was the first attempt towards supervised learning-based lifetime estimation. Their authors trained a shallow Multi-Layer Perceptron (MLP) with two hidden layers, where histograms of photon count having 57 bins are directly fed to the network as input features. The assumed fluorescence decay model is bi-exponential. In [79], a DL-based lifetime estimation was developed via a 3D CNN architecture. The authors considered the input of the network as a 3D tensor of (*x*, *y*; *t*). Their network topology consisted of an initial pedestal branch for extracting temporal correlations and three subsequent parallel branches for spatial filtering and regression purposes. Outputs of the network encompass three 2D maps including images of amplitude and two lifetime components of a bi-exponential fluorescence decay model. Training data of the CNN was generated from synthesised temporal histograms inserted into white-pixel locations of a binarised version of the well-known MNIST character recognition dataset. Zickus *et al* [77] and Xiao *et al* [80] have suggested other FC NN and CNN architectures for lifetime estimation, where their specifications are summarised in table 1. Other authors used similar techniques [94–96]. E.g., a FC NN with the aim of reducing complexity than deep schemes and improving estimation efficiency (i.e., bias and variance) was developed in [94]. It extracts four features from each temporal histogram (including 2 phasor parameters) and approximates a bi-exponential model, hence constituting a 4-input, 11-hidden layer (6 neurons per layer), and 3-output architecture. Similar CNN-based architectures in [95, 96] were proposed for reconstructing both intensity and lifetime images from a compressive sensing-based single-pixel FLIm acquisition system [97]. Chen *et al* [78] and Taimori *et al* [74] employed game theoretic approaches to deal with challenging scenarios of photon-starved (50 p/p) and Low Photon Count (LPC) regimes. The former exploits a Generative Adversarial Network (GAN) architecture at its heart to be able to generate high-quality temporal data by using the learning distribution of histogram of photon counts generated from a set of real samples via a generative model training. The latter proposed a Robust RLD algorithm that models the lifetime estimation problem in the presence of both noise and blurring perturbations as a signal recovery. It represents the histogram of photon counts as a multi-resolution framework, where perturbed measurements are denoised via adaptive smoothing mechanisms.

Here, as a critical conclusion, ML-based lifetime estimation versions substantially improved standard metrics such as Mean Absolute Error (MAE), Mean Squared Error (MSE), bias and variance of the fluorescence lifetime estimates in comparison to custom baseline techniques like RLD [81, 82], CMM [85], LS [83, 84] and MLE [87, 88]. Among all, very promising results are seen for the game-theoretic approaches, especially on LPC regimes [74, 78] as a sign of further attention. Nonetheless, challenges yet exist for the realisation of state-of-the-art methods in industrial microscopes and endomicroscopes. For instance, handling of the problem on a very wide range of physical parameters (e.g., imaging samples ranging from sub-nanosecond to hundreds of nanosecond lifetimes) in a completely controlled manner, fully automating and hardware embedding are issues that will demand more research and development in future towards mature lifetime estimators.

### 2.7. Lifetime signal to noise ratio

In fluorescence, the lifetime is denoted by *τ*. The fluorescence lifetime error (standard deviation) Δ*τ* and SNR as:

\begin{eqnarray*}\mathrm{Lifetime}\,\mathrm{SNR}=\tfrac{\tau }{{\mathrm{\Delta }}\tau }\end{eqnarray*}

ultimately determine the sensitivity and image contrast achievable in FLIm. To apprise SNR, the measure $F-\mathrm{value}\triangleq \tfrac{\sqrt{N}{\mathrm{\Delta }}\tau }{\tau }$ is also employed in the literature [98], in which the parameter *N* denotes the number of detected photons and *F* − value ≥ 1. In 1992, Köllner and Wolfrum [99] addressed the important question of how many time-domain photon detections are required to perform measurements of fluorescent lifetime with a given accuracy. The required number of photons for a desired variance is:

\begin{eqnarray*}N\geqslant \tfrac{{{var}}_{1}(\tau )}{{desired}\,{var}(\tau )}\end{eqnarray*}

where *var*_1_(*τ*) is the variance of fluorescence lifetime *τ* when the signal is only one photon. For a mono-exponential decay, the variance can be written in an explicit form as:

\begin{eqnarray*}\begin{array}{l}{{var}}_{N}(\tau ,T,k)=\tfrac{1}{N}{\tau }^{2}\tfrac{{k}^{2}}{{r}^{2}}[1-\exp (-r)]\\ \times {\left(\tfrac{\exp \left(\tfrac{r}{k}\right)[1-\exp (-r)]}{{\left[\exp \left(\tfrac{r}{k}\right)-1\right]}^{2}}-\tfrac{{k}^{2}}{\exp (r)-1}\right)}^{-1}\\ =\,\tfrac{1}{N}{{var}}_{1}(\tau ,T,k)\\ =\ \tfrac{1}{N}{\tau }^{2}{{var}}_{1}(r,k)\end{array}\end{eqnarray*}

where *T* is the length of the measurement window, *k* is the number of channels in the window and *r* = *T*/*τ* is the number of lifetimes in the measurement window.

The standard deviation of lifetime for mono-exponential decays can be written as:

\begin{eqnarray*}\begin{array}{l}{\mathrm{\Delta }}\tau ={\sigma }_{N}(\tau ,T,k)\\ =\sqrt{{{var}}_{N}(\tau ,T,k)}\\ =\sqrt{\tfrac{1}{N}{\tau }^{2}{{var}}_{1}(r,k)}\end{array}\end{eqnarray*}

The fluorescence lifetime normalised error becomes [100]:

\begin{eqnarray*}\tfrac{{\mathrm{\Delta }}\tau }{\tau }=\tfrac{1}{\sqrt{N}}\sqrt{{{var}}_{1}(r,k)}\end{eqnarray*}

The fluorescence lifetime SNR is simply the inverse of equation (9). The Kollner-Wolfrum formulae (equation (6)) demonstrate that lifetime errors of less than 10% can be achieved with hundreds of photons or less and provide a useful model for tuning experimental parameters such as the number and optimal width of time channels, total acquisition time range and fluorophore lifetime contrast.

**Figure 5.**
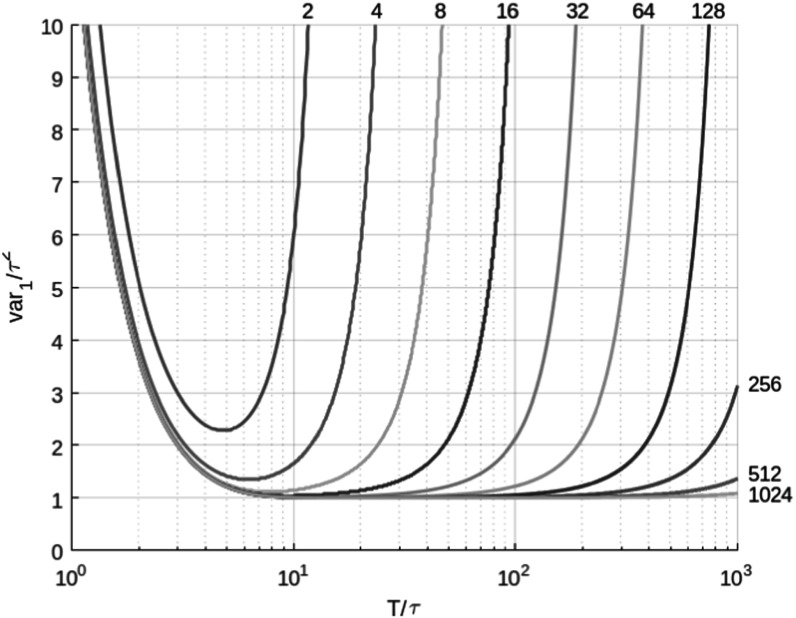
The normalised variance of *τ*, *var*_1_/*τ* for mono-exponential decays as a function of T/t for different numbers of channels. T is the total width of the measurement window. The normalised variance is proportional to the number of photons needed for a certain accuracy [99]

In both time gating and TCSPC, two channels/bins are often utilised and, as shown in figure 5, the variance for this simplified case is seen to be greater and more sensitive to time-range than in cases where more channels are utilised. A lifetime error analysis for the two-channel case has been carried out by Ballew and Demas [81]. Philip and Carlsson [98] investigated the fluorescence lifetime imaging SNR in the context of frequency domain lifetime measurement methods and carried out comparisons with time domain techniques. On-chip fluorescence lifetime algorithms and SNR were investigated by Li *et al* [101].

From lifetime images and from repeated fluorescence lifetime SNR measurements, lifetime probability distribution functions can be derived. The degree of overlap in these distributions between different fluorophores ultimately determines the efficacy of lifetime as a signature of normal or diseased tissue states. When lifetime is used as a diagnostic test, the complete separation of two fluorescence lifetime distributions implies a perfectly discriminating test, while complete overlap implies no discrimination [102]. From the distributions, we can compute Receiver Operating Characteristic (ROC) which enables medical image data to classify patients as positive’ or negative’ with respect to any particular disease [103]. ROC analysis of diagnostic models has been utilised in ML-assisted fluorescence lifetime imaging endomicroscopy ex-vivo lung cancer studies by Wang *et al* [104] and in studies by Chen *et al* [105] aimed at distinguishing basal cell carcinoma from actinic keratosis and Bowen's disease.

## 3. Fluorescence lifetime image analyses

### 3.1. Fluorescence lifetime image registration

**Figure 6.**
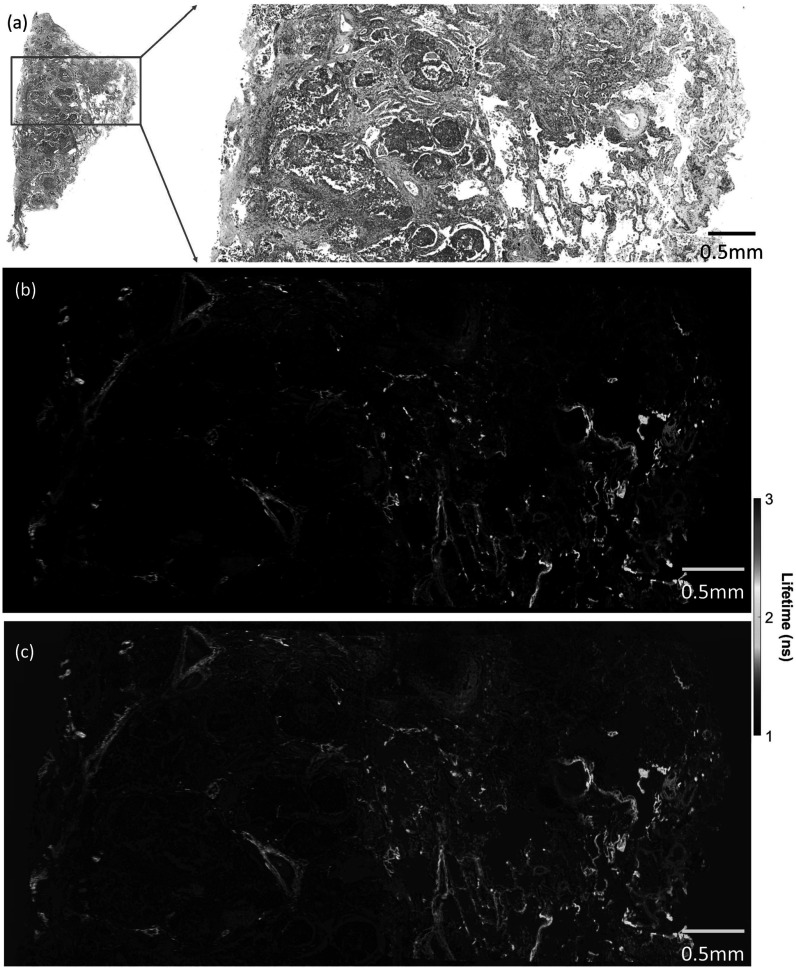
Co-registration of a FLIm image (b) with the corresponding histology image (a) to enable cellular-level correlation of FLIm and histology images for cellular-level analysis. (c) is the blending of the co-registered FLIm and histology images [110]. Annotated by an experienced pathologist, the histology image (a) contains both normal (right part) and cancerous areas (left part), along with the normal-to-cancer transition at the centre. Accordingly, the FLIm image (b) demonstrates the decrease in label-free autofluorescence lifetime values from normal to cancerous areas.

Image registration is an important task in medical image analysis. It facilitates the matching and alignment of images from different modalities or different time periods in the same modality, aiming to provide the end-user with richer diagnostic information [106, 107]. Currently, good results can be achieved with the many registration algorithms proposed in the literature. However, very limited studies have looked at the registration problem in microscopic imaging or FLIm, while most of the algorithms are considered to be very specific to certain clinical applications or datasets. As described in section 2, lifetime contrast is based on the averaged lifetime derived from statistical methods, for example, histogramming of lifetime images [30, 108]. Unfortunately, the statistical derivation is unable to reveal cellular-level characteristics of fluorescence phenomena under investigation. An insightful understanding of the investigated tissue requires the correlation at the pixel level of the FLIm images with a reference image, such as a histology image, which can be achievable through co-registration. As a result, conclusive decisions can be achieved for various purposes, for example, cancer detection [30, 36], tumour margin detection [109], or cellular-level cancer characterisation [110]. Figure 6 shows an example of the co-registration of a FLIm image with a Haematoxylin and Eosin (H&E)-stained histology image, where pixel lifetime can be correlated with tumour components in the histology image by detailed annotations. However, the co-registration remains challenging due to the different nature of FLIm and histology images [58].

Since FLIm images contain both intensity and the corresponding lifetime images, both modalities could facilitate the co-registration. Intuitively, the intensity seems more suitable than the lifetime for the task as both intensity and histology images present fluorescence concentration distribution via optical scanning. In contrast, lifetime images are usually homogeneous due to the intensity independence of lifetime—raw lifetime images tend to have a flat nature. It is worth mentioning that a common practice to overcome the homogeneous presentation is to saturate a lifetime image with its corresponding intensity image so that both structural and spatial distribution in FLIm images can be explicitly illustrated [12, 30, 108]. In addition, spectral FLIm images at various emission wavelengths are often visually different since certain structural features may only emit at a particular range, which also deteriorates the effectiveness of the co-registration [12, 111]. Due to the complexity of the staining procedure, artefacts are often introduced to histology images, such as colour variations caused by the differences in staining and the scanners [112], distortion of tissue structure [113], or tissue contamination [114]. All these factors contribute to the challenges of co-registration.

Despite the importance of co-registration, little effort has been dedicated explicitly to it. Generally, co-registering FLIM and histological images can be performed on fixed or unfixed tissue. Since the underlying structure is unchanged, the co-registration of the images on fixed tissue is relatively straightforward. In this case, non-rigid registration could be applied directly. Bird *et al* [115] applied a landmark-based semi-automatic registration to spatially align spectral FLIM and H&E-stained histology images acquired on H&E-stained tissue for lung cancer diagnosis. Wang *et al* [110] used a widely-used regression-based approach to estimating the homography matrix for the co-registration of full-spectral FLIM and H&E-stained histology images to extract lifetime signatures of lung cancer. When it comes to unfixed tissue, the situation becomes more complicated as, in most cases, the spatial structure has changed, and advanced technologies may be required to deal with the distortion. For example, Unger *et al* [116] employed an indirect method to align *ex vivo* tissue with H&E-stained histology images. In their report, the histology images were first correlated with the corresponding white-light images of the tissue using a hybrid method containing landmark-based and deformable registration, and the relevant lifetime images were mapped to the histology images based on the white-light images.

As far as biomedical image registration is concerned, the success of DL technologies has significantly advanced registration performance, where both supervised and unsupervised techniques have achieved remarkable results for rigid and deformable registration [106, 107]. However, their applicability to FLIm image registration may not be straightforward. The reasons are multifold. FLIm and histology images usually contain sub-billion pixels. The direct application of the existing methods requires a significant amount of computational resources, which are not always available. As a result, patch-level registration may be a better solution, such as [110, 113]. Meanwhile, DL-based registration usually requires a significant number of images for training and validation, which is not always available, particularly for whole-slide images. In addition, obtaining ground truth in this domain remains challenging, which presents obstacles to the application of matured DL-based registration approaches to FLIm images. Nevertheless, some proven registration techniques for microscopic images could inspire the development of this area. Jiang *et al* [117] applied a hierarchical strategy to a kernel density estimator so that H&E and immunohistochemical (IHC)-stained WSI images can be robustly correlated. Ge *et al* [118] proposed an unsupervised DL model to align histological images generated using different staining techniques, where multi-scale structural features were retrieved to guide the model to generate an optimal deformation field.

### 3.2. Segmentation, detection and tracking in fluorescence imaging

Segmentation, detection, and tracking of biological and chemical substances are pivotal for a comprehensive semantic analysis of FLIm data. Such capabilities underpin critical applications like cancer analysis and drug-target engagement [119–121]. Examples include, segmenting, detecting, and tracking microscopic images of viruses (e.g., SARS-CoV and its variants [122]), bacteria like E. coli and Staphylococcus aureus [123], as well as various types of cells [124]. Essentially, the aim is to identify and quantify objects of interest to provide an enriched image semantic analysis.

Examples of such methods within FLIm include the automatic segmentation of cellular FLIm images. This technique is inspired by a multiresolution community detection-based network segmentation approach. It focuses on distinguishing segments based on their average lifetimes relative to the background of the FLIm image [125], which underlines the role of precise segmentation in decoding cellular structures and interactions.

The journey from conventional image processing to machine and deep learning in image segmentation is noteworthy. A recent innovation combined machine learning-based interactive image segmentation with supervoxels and clustering. This fusion aids in automatically identifying similarly coloured images across vast datasets [126]. It addresses challenges like colour variability in biological and medical images, paving the way for more efficient interactive segmentation on larger datasets.

In terms of object classification, binary segmentation focuses on differentiating background from foreground at a pixel level, helping to identify the location and concentration of objects, whereas tracking interprets the objects’ movement and path [127]. Historically, techniques used for segmentation, detection, and tracking can be categorized into three domains:

(1) Traditional digital processing methods using Gaussian Mixture Model (GMM), such as Otsu’s thresholding [128], and other methods such as mean shift [129], and the Kalman filter [130].
(2) Feature engineering-based training approaches as seen in the works of Viola and Jones [131] and Kalal *et al*. [132].
(3) Techniques centered arfound DL [133, 134].

**Figure 7.**
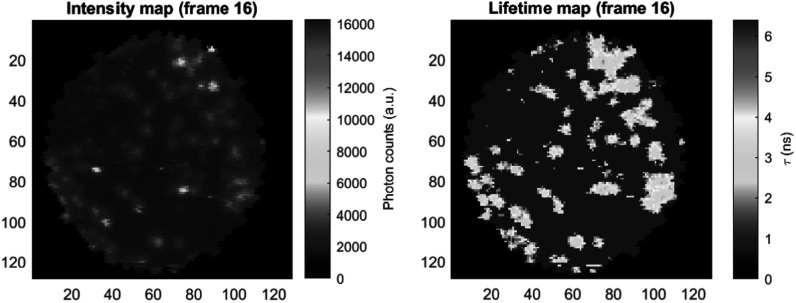
An illustration of microbeads’ objects in saline on both intensity and lifetime maps for segmentation, detection and tracking purposes.

In clinical microscopy imaging, precise object recognition can be intricate due to problem-specific factors. For instance, detecting specific bacteria [123] is often subject to expert opinion, making it more challenging than problems with clear visual cues. In the field of fluorescence imaging, many researchers have utilized computer vision techniques specifically for steady-state systems, as indicated by [135]. However, there is a growing emphasis on transitioning to the time-resolved capabilities provided by FLIm. Such a transition offers valuable insights into microorganisms, ranging from their fundamental structures to their ecological interactions. The essence of this idea is evident in the object mapping within FLIm systems:

\begin{eqnarray*}f:({o}_{1},...,{o}_{m})\mapsto ({o}_{1}^{{\prime} },...,{o}_{n}^{{\prime} }),\end{eqnarray*}

where the object *o*_*i*_, *∀* *i* = 1,...,*m* in an intensity map corresponds to the object ${o}_{j}^{{\prime} },\forall j=1,\cdots ,n$ in its lifetime match by the mapper *f*. As a tangible illustration of the concept of the mapping, figure 7 shows, at a frame sequence, how polymer microbeads (as objects of interest) in saline appear on both intensity and lifetime maps. As a result, the segmentation, detection and tracking tasks can be tested on the complementary lifetime map [135].

Moreover, the manual analysis of imaging data is labor-intensive, subjective, and resource-heavy, making automation crucial. Koujan *et al* [154] offered an automated method to segment pulmonary Optical Endo-microscopy (OEM) imaging data based on their diagnostic importance, leveraging local binary patterns, the Support Vector Machine (SVM) classifier, and a one-versus-all strategy for improved accuracy. In bacterial detection, both supervised and unsupervised methods find applications . Research like [155] and [156] used supervised models for bacteria detection in microscopy images. In contrast, unsupervised methods, like the Bayesian approach for detecting microorganisms in pulmonary OEM imaging, treat bacterial objects as outliers added to actual pixel values disturbed by Gaussian noise [157]. Another approach divided images into overlapping patches, transforming bacteria detection into a minimization problem [158].

For other microorganisms’ shapes can often be represented as 2D and 3D mathematical models. For instance, Zamani *et al* [124] introduced a mechanism for detecting human cells like HT-29 in the blood. They viewed cell detection as ellipse recovery, using a 2D CNN to estimate an elliptical shape’s parameters.

Drug discovery is another area where the detection of biological phenomena using FLIm is deemed useful. One of the key elements across the drug discovery pipeline is the accurate quantification of protein-protein interactions, also referred to in drug development as drug-target engagement [159]. There are different methods to measure these interactions, such as surface plasmon resonance, nuclear magnetic resonance spectroscopy, and many more [160]. However, these methods are limited and often require protein purification, which may alter the true functionality of proteins and sample preparation [160]. In recent years, the detection of FRET using FLIm technology has provided a robust imaging modality for drug discovery, enabling a reliable readout of sub-cellular information while significantly reducing photo-toxicity levels [159]. For example, Ochoa *et al* [161] reported the development of an Macroscopic Fluorescence Lifetime Imaging (MFLI) optical imaging system for the quantitative measurement of *in vivo* drug-target interaction. The system reported in the study acted as a direct lifetime sensor of FRET in live animals. Additionally, a recent study by Yang *et al* [162] highlighted the potential of live-cell image-based ML strategies in reducing variability in Pluripotent Stem Cell (PSC) differentiation systems, which can be instrumental in supporting recent FLIm and ML advancement in drug discovery, disease modeling, and regenerative medicine. The study emphasized the role of artificial intelligence in guiding and optimizing PSC differentiation, offering insights into the differentiation process for functional cell manufacturing in biomedical applications.

### 3.3. Fluorescence lifetime imaging knowledge extraction and categorisation

#### 3.3.1. Machine learning uses cases in FLIm

Fluorescence-based medical imaging has proved to be effective for cancer discrimination for decades [36, 43, 163, 164]. However, Marcu [36] found in 2012 that the majority of those studies had been made on Fluorescence spectroscopy [165–168]. Meanwhile, research on FLIm was still relatively new at that time. However, ten years later, new studies have been published about FLIm and cancer [169–171], with the majority coming from a small number of research groups, thus limited in diversity.

ML has not been extensively used for images features extraction on FLIm data, such as disease detection or tissue segmentation. However, the number of publications has been steadily increasing since 2018, as shown in table 2. From the literature, we can differentiate the following uses-cases: image-level classification, which assigns one label to an entire sample or region, and pixel-level classification, which gives a label to every pixel. The latter can then be used either for semantic segmentation, as each pixel is labeled, or for image-level classification by averaging the labels across all pixels. Both uses-cases can be applied to discriminate any number of classes such as cancer, pre-cancerous, adipose tissue, etc. The most common type of classification, binary classification, can be used, for example, to differentiate cancer (positive) from everything else (negative) without paying attention to specific details of the tissue. More recent studies have investigated ML cells classification, some of them being listed in table 2 [148–153]. They classify the type of cells or their states, such as activated and quiescent for T-cells.

**Table 2.** Summary table of all ML paper reviewed in this section. The region used corresponds to the input fed to the model, either one pixel or a region (i.e. a group of pixels, like an image). For the pixel level, the classification result can be averaged across all the pixels of one sample. The majority of cell research uses the lifetime captured on one cell.

Author	Year	Medical effort	Detail	Region used	ML type	Detail	*in vivo*
Bianchetti *et al* [41]	2021	cancer	breast	clustering			
Phipps *et al* [4]	2017			pixel	classification	Binary (1 versus N)	
Unger *et al* [5]	2020			point	classification	3 classes	
					detection	Binary	
			
Jo *et al* [136]	2018		oral	pixel (average)	classification	Binary (1 versus N)	*in vivo*
Marsden *et al* [109]	2020			point	detection	Binary	*in vivo*
				point (average)			
Caughlin *et al* [137]	2021			pixel (average)	classification	Binary (1 versus N)	*in vivo*
Duran *et al* [138]	2021			pixel (average)	detection	Binary (1 versus N)	*in vivo*
			
Cosci *et al* [139]	2016		skin	(5) pixel (average)	detection	Binary	*in vivo*
					classification	3 classes	
Chen *et al* [105]	2019			subregion	classification	Binary	
						Binary (1 versus N)	
Yang *et al* [140]	2020			region	classification	Binary	
Romano *et al* [141]	2020			region	detection	Binary (1 versus N)	*in vivo*
Vasanthakumari *et al* [142]	2022			region as feature	detection	Binary (N versus N)	*in vivo*
			
Wang *et al* [119]	2020		lung	region	detection	Binary	
Wang *et al* [104]	2021			region	detection	Binary	
Gu *et al* [143]	2014		cervical	region (mean/std)	detection	Binary (1 versus N)	
					classification	Binary (N versus N)	
					detection	Binary (1 versus N)	
			
Gu *et al* [144]	2015			region	detection	Binary (1 versus N)	
Sahoo *et al* [145]	2018			region	classification	Binary	
Ji *et al* [146]	2022			region	classification	Binary (1 versus N)	
			
Weyers *et al* [3]	2022		oropharyngeal	point	detection	Binary	*in vivo*
			
Butte *et al* [147]	2011		brain	single point	classification	4 classes	*in vivo*
Walsh *et al* [148]	2020	cell	T-cell	subregion (cell)	classification	Binary	
						4 classes	
			
Dunkers *et al* [149]	2021		S. mutans	pixel (average)	classification	3 classes	
Qian *et al* [150]	2021		Stem cell	subregion (cell)	classification	Binary	
			
Cardonna *et al* [151]	2022		T-cell & Cancer cell	subregion (cell)	classification	4 classes	
			
Neto *et al* [152]	2022		macrophage	subregion (cell)	classification	Binary	
			
Kröger *et al* [153]	2022		macrophage	subregion (cell)	classification	Binary (N versus N)	*in vivo*
						Binary (1 versus N)	
						Binary (1 versus N)	
						Binary	

Marsden *et al* [10]	2021	parathyroid/thyroid		point	detection	Binary (1 versus N)	*in vivo*
				point (average)	detection		

For binary classification, N refer to multiple class grouped into one, while 1 refer to only one label. E.g: Phipps *et al* [4] do Binary (1 versus N) as they classify cancer tissue (1 label) vs other tissue (fibrous and adipose grouped together, so 2 label grouped as 1).

Classification with pixel as region used refer to pixel-by-pixel classification, used for segmentation. It is not listed as semantic segmentation, since only one pixel is used each time as input.

In their review, [24] reported the absence of DL-based classification research on FLIm, but concluded that it would play a critical role in the future. This observation is reinforced when considering the growth of ML and DL techniques in the last decades, the accessibility of programming languages like Matlab [172] and Python [173], and frameworks such as Scikit-Learn [174].

However, as the learning curve flattens, research may be carried out without the proper methodology, and confusion can also be found in published results. Moreover, ML is also used as an analysis tool, to prove the effectiveness of FLIm data for a particular task, but fewer efforts are made after this to actually develop custom ML, state-of-the-art models.

#### 3.3.2. Performance evaluation in ML tasks applied to FLIm

ML model evaluation is performed by comparing metrics generated from their outputs. Those metrics are chosen depending on the type of model and the specific of the problem being solved. Regression metrics have been discussed in section 2.6. Classification and segmentation use a subset of overlapping metrics, commonly used in a wide variety of domains such as information retrieval, statistics or detection theory. Those metrics are based on the four possible outcomes of a binary prediction, which features two labels, positive (P) and negative (N), as well as two possible predictions, positive (PP) and negative (PN): True Positive (TP) (P and PP), True Negative (TN) (N and PN), False Positive (FP) (N and PP), False Negative (FN) (P and PN), which can be extended to multi-class classification by choosing one class as positive and grouping all the others as negative. For example, consider the three possible results of a test: healthy, pre-cancerous, cancerous. By choosing cancerous as ‘positive’ and the rest as negative, the number of TP or TN can be deduced. Based on those outcomes, several metrics can be derived, such as sensitivity $\left(\tfrac{{TP}}{P}\right)$, specificity $\left(\tfrac{{TN}}{N}\right)$, precision $\left(\tfrac{{TP}}{{PP}}\right)$, accuracy $\left(\tfrac{{TP}+{TN}}{P+N}\right)$,F1-score $\left(\tfrac{2{TP}}{2{TP}+{FP}+{FN}}\right)$ and other [175].

In general, accuracy is considered a key metric for classification, when the different classes are balanced [176]. Otherwise, if one class is under-represented, the accuracy will be biased towards one or multiple classes. However, in some situations, such as medical diagnosis, the sensitivity and/or specificity may be more important because the detection probability of the model is evaluated [175]. The dice score, or F1-score, is an important metric for semantic segmentation [177], but specific metrics derived from the segmented object may be used. For example, if the objects can be counted or if they can be connected, such information can be transformed into metrics. Pixel-by-pixel semantic segmentation classifies one pixel at a time, without knowledge of the other pixels. This type of segmentation is close to classification, and thus similar metrics are often used. On the other hand, region-level segmentation, which takes an image as input and outputs an image, often uses the Jaccard index in addition to the dice score.

#### 3.3.3. Machine learning modalities: model

Random Forest (RF) and SVM are the two most used models in the studies listed in 2. Extreme Learning Machine (ELM) has been used twice [144, 179]. DL implementations in FLIm range from MLP with dense layers, used alongside RF and SVM without distinction, to custom implementations of pre-trained CNN. However, DL has been scarcely used.

More complex ML architecture started to be created for FLIm. An autoencoder joint with a dense neural network has been developed by [137]. It works directly on the decay curve and does not use hand-crafted features (such as lifetime or phasor plot value). Wang *et al* [104] developed a custom CNN architecture by replacing some layers and building it on top of the classical ResNet [180] architecture.

CNNs are now widely used, and pre-trained networks are readily available online. Architectures such as ResNet, Inception [181] or EfficientNet [182, 183] can be found in many DL libraries and frameworks, and will provide researcher classification capacity far superior to simpler machine learning models. Through fine-tuning [184], those models can be easily trained without needing as many resources as being trained from scratch, and could provide better results than simpler ML models. Fully-convolutional NN such as UNet [25], or its state-of-the-art variants like UNet++ [185] and UNet3+ [186], should be preferred for segmentation tasks to pixel by pixel classification models. Those CNNs can take advantage of spatial feature information and are specifically designed for segmentation task, with a focus in medical imaging.

Generally speaking, DL models will take as input an entire multi-spectral FLIm image. In the case of intensity and lifetime, images can be either concatenated to be fed as one in a NN, or two networks can be used for feature extraction, before being concatenated and then fed into the classifier part. Alternatively, intensity and lifetime can be mixed, similarly to what is shown in 2.

#### 3.3.4. Machine learning modalities: features

The FLIm features that were captured in each study are listed in table 4. They were either directly used as input or processed and/or went through feature selection. Principle component analysis (PCA) has sometimes been used to reduce the dimensionality of the images, projecting the data into a new space but losing the spatial information [119, 145]. Duran-Sierra *et al* [138] used sequential forward selection to find out which features would be the best for classification. Other methods have been used to evaluate the ‘weight’ or the impact of each feature, e.g removing each feature from the model’s input and observing the classification results [150]. However, the final model they presented still uses all collected features without excluding any. They were not the only ones to evaluate subsets of feature, usually handmade subsets such as ‘intensity only’ or ‘lifetime only’ [136, 138, 148, 152].

*Ex vivo* experimentation is more common because it is easier, especially for cell studies which can be cultured. For tissue, imaging it *in vivo* means bringing the hardware into a medical setting which generally implies significant restrictions that are harder to meet during an experimental study. However, a few groups have been working on *in vivo* applications (Jo [136, 137, 141, 142]), (Marcu [3, 10, 109]), Cosci *et al* [139] and Butte *et al* [147]. Except for Butte *et al* who worked on brain cancer, all the other diseases investigated targeted the skin or the head and neck region, such as oral cancer. The data gathering methods are split between non-invasive analysis of the tissue, e.g directly on the skin for skin cancer, and invasive method, with the FLIm data being captured during a biopsy before the tissue is removed. Only [109] used both *ex vivo* and *in vivo* samples and obtained better results *in vivo*, with 86% and 72% accuracy for *in vivo* region level and point level classification, against 78% and 67% for *ex vivo*. With only one study for comparison, it is impossible to draw any conclusion on the usability of ML for *in vivo* live recognition of tissue.

#### 3.3.5. Challenges and issues in machine learning

As described earlier, a model should be trained and then tested on different data. Alternatively, if the number of samples is too low, Cross-Validation (CV) can be used, by partitioning one dataset in *N* parts, training a different model on *N* − 1 partition and testing it on the remaining one, multiple times [175]. CV is normally used for optimising the hyper-parameters of a model but can be used for testing as we described as well. However, testing methods are not meant to be compared and should not be considered hyperparameters. Yang *et al* [140] used bootstrapping, hold-out and K-fold CV (K-CV) and compared the accuracy obtained. Besides wrongfully comparing the methods, the difference in the value of their metrics is very wide, hinting at some issue with over-fitting or in the distribution of their test set.

In the same manner, the training and testing sizes are not meant to be hyper-parameters, as the model would be optimised to improve the testing set results, which is equivalent to over-fitting to it rather than simply use it for evaluation. For example, [148] used different threshold split and handpicked the best result, which can lead to confusion on how well their model is performing. Similarly, it is not clear which validation method was used by Sahoo *et al* [145] who seems to mix CV and hold-out.

Incorrect uses of vocabulary are generally harmless but, in some cases, can lead to some confusion. For example, [148] regularly exchanges ‘accuracy’ and ‘Area Under the Curve (AUC)’ when referring to the ROC curve. This error can also be found in other papers such as [5, 152] or [144]. While they are usually understandable with the context, they can create misunderstanding, especially if both metrics are used and their results differ.

In their paper, Sahoo *et al* [145] defined a custom formula ‘combining’ sensitivity and specificity, which led to results of 100% for each value, but an accuracy of only 84% which is not possible. Metrics such as those defined earlier are standards that are used by everyone and are not meant to be modified. to allow for a fair comparison.

#### 3.3.6. Challenges and issues in FLIm

The multiple exterior factors influencing the intensity and lifetime value, such as biological difference (pH, oxygen level, type of tissue imaged, ...) or spectroscopy parameters (absorption and emission wavelength, numbers of photons, ...) make it hard to compare different FLIm studies. As cited in Subsection 2.4, different systems exist: time and frequency domain. Additionally, custom homemade FLIm devices are used by a large number of groups, such as [105, 109, 136] to only cite a few, increasing the disparity in the data collected.

**Figure 8.**
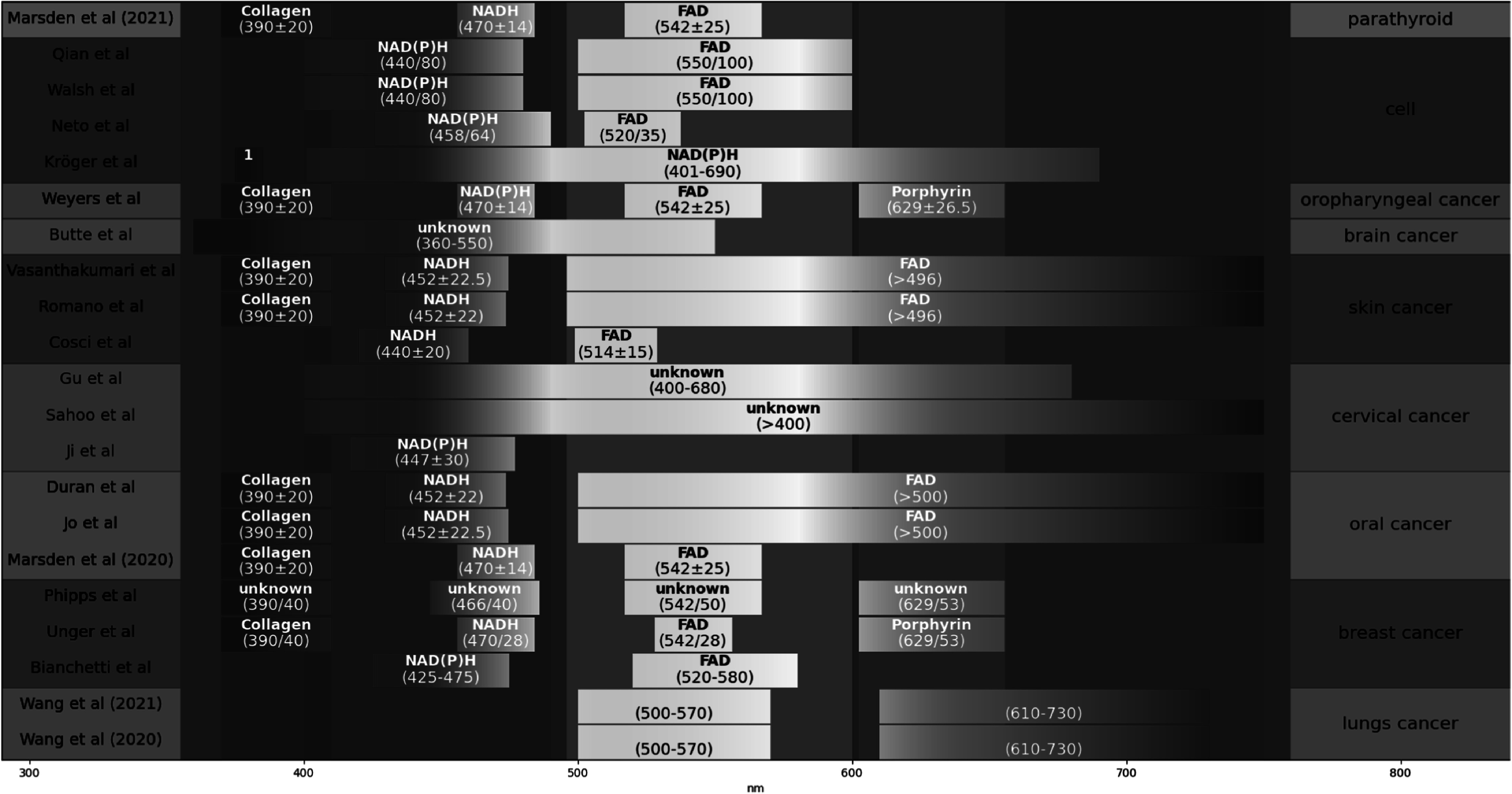
Visualisation of the channel bandpass/filter used on the emission wavelength. The target is listed when it has been mentioned. 1: The first filter from 375 nm to 385 nm was used to detect the second harmonic generation signal.

When exciting tissues, researchers are looking to target particular enzymes or probes, such as FAD or NADH. The choice of the target may be impacted by the type of tissue or cells. For example, Walsh *et al* [148] decided to use NAD(P)H after evaluating a sample of T-cell. Those different enzymes do not have the same properties and may react differently depending on the environment but also the excitation wavelength, impacting their emission wavelength. Figure 8 shows a visual representation of the emission wavelength captured, associated with the fluorophores (when listed). Different sources are cited when it comes to the filter used, all listed for each group in table 4. In the majority, groups tend to cite papers they have written previously, such as Marcu group [4, 5, 109] citing Yankelevich *et al* [187], Skala group [148, 150] citing Skala *et al* [43] or Jo *et al* [136] citing Cheng *et al* [188] to justify the channel used.

Moreover, even if they are looking at the same FLIm information, researchers may not use the same features as input for their models. They may use the intensity, the lifetime, the average lifetime, or other specific variables, such as the Laguerre variable, or the Shannon entropy, as seen in table 4. Thus it makes it harder to compare the results of two different studies even when they are from the same group.

#### 3.3.7. Challenges and issues with metrics

Metrics are used to evaluate a model, to highlight its performance but also its flaws. Their choice, as described earlier in section 3.3.2, should be tailored to the problem tackled. For the common case of cancer detection seen in table 2, with cancer considered ‘positive’, a false negative have a greater impact than a false positive, as missing a cancer case during diagnosis carries more risks than diagnosing it to an healthy person. Therefore, a metric such as sensitivity needs to be used. However, it can not be used by itself, as classifying every sample as positive would lead to a perfect sensitivity, but a very inaccurate model. It should be paired with another metric such as specificity, to evaluate how well the model can recognise the disease. The distribution of the class should also be considered to not bias the metrics [175]. For this example, Unger *et al* [5] used the Matthew Correlation Coefficient (MCC). It has previously been described as a metric which properly takes care of unbalanced dataset [190], as the one Unger *et al* has, but this has been challenged recently [191]. Since Unger *et al* did not provide other metrics than MCC and ROC AUC, and no other studies in table 2, it is impossible to compare their results. This would be fine if they are the only one featuring an imbalanced dataset, but if other studies choose metrics that fit only with balanced data, then two issues are created: improper choices of metrics not reflecting the real performances of the model, and the impossibility of comparing the results with studies using proper metrics. The ROC AUC is also often used, when applicable, but, as with all metrics, it should not be used alone as it measures the model’s capacity to rank the samples rather than its capacity to classify them. It can be used to evaluate the trade-off between specificity and sensitivity. While imperfect, accuracy is still a pretty commonly used metric, especially in research to improve ML models, where many datasets are balanced so that the metric is still relevant for problem evaluation, Therefore, it should also be included when possible. Regarding segmentation, metrics commonly used, such as the dice score and Jaccard index, should be preferred to accuracy, which can be easily biased by an imbalance in the labels. However, those metrics are dependent on the choice of the ‘positive’ class and thus need to be properly set to target cancer or other diseases when using binary classification.

#### 3.3.8. Challenges and issues with information sharing and reproducibility

Apart from the lack of standardization and common elements between different research ML and FLIm research, another issue is the limited information about the ML methodology which hinder the reproducibility of the work. It is sometimes hard to understand which features were used or how they were obtained. Information about the data format is not always explicitly provided, which also applies to information about the dataset, as seen in tables 3 and table 4. Even when included, the information will often be hard to understand for someone not knowledgeable about FLIm, and inter-disciplinary researcher may struggle to properly extract information from those studies. Information such as the number of samples, the studied area or the data format, and more general details provided in tables 2–4, should be clearly stated and understandable for everyone.

**Table 3.** Performance summary of the studies cited in figure 8, sorted chronologically. All models and validation methods have been listed, and the most important experiment’s results has been singled out for each study, with its specification in the last column, and the model used emboldened.

Author	Year	Validation	Model	ACC	SE	SP	AUC	Specification
Butte *et al* [147]	2011	LOOCV	LDA	71.43%	47.06%	94.64%		Temporal + Spectral SE/SP: HGG
Cosci *et al* [139]	2016	10-fold CV	KNN (K=5)	90%	92.8%	88.8%		SE/SP: healthy vs other
Phipps *et al* [4]	2017	LOPOCV	SVM (rbf)	97.8%	100%	96.90%		balanced cancer vs other
Jo *et al* [136]	2018	LOPOCV	QDA	89%	95%	86.79%	0.91	FLIm features
Unger *et al* [5]	2020	LOPOCV	RF		88.78%	93.14%	0.96	tumour vs no tumour
Marsden *et al* [109]	2020	LOOCV (tongue/tonsil only). Test on *other* with RF	**RF**, SVM (rbf), 1D-CNN		86%	87%	0.88	*in vivo*, region
Walsh *et al* [148]	2020	Split train/test	**LogReg**, RF, SVM				0.95	type and activation
Wang *et al* [119]	2020	LOPOCV (3 times) train=(90% / 10%) train/validation	2D-CNN 3D-CNN	86.5%	89.5%		0.858	**3-channel DenseNet121**
Romano *et al* [141]	2020	Split train/test 75% / 25%	LDA	73%	88%	67%	0.79	intensity and lifetime
Dunkers *et al* [149]	2021	Out of bag no validation	RF	95.91%				PBS bufffer, lifetime and phasor variable
Wang *et al* [104]	2021	One patient out train=(90%10%) train/validation	Custom 3D-CNN	84.9%	80.95%		0.882	**MSCD-ResNet50 preserved complexity**
Qian *et al* [150]	2021	train/test set train=(80% / 20%)train/validation	**LogReg**, RF, SVM	>85%			0.9085	all variable logistic regression
Marsden *et al* [10]	2021	LOPOCV	NN, **RF**, SVM		100%	93%		region level
Duran *et al* [138]	2021	7-fold CV (train) Best model on test	NN, SVM, RF		78%	61%	0.81	ensemble
Weyers *et al* [3]	2022	Pre-trained then tested	RF		96%	89%	0.9	mean over patients
Neto *et al* [152]	2022	10-fold CV	RF				0.944	all 2p FLIm variables
Vasanthakumari *et al* [178]	2022	LOPOCV	QDA	88.33%	84.21%	90.24%		Phasor + intensity + lifetime variables
Ji *et al* [146]	2022	Train: 151 Cancer/CNI +217 normal Test: Images from 48 patients	K-means as classifier		90.90%	100%	0.95	*τ*_avg_ + *α*_2_
Kröger *et al* [153]	2022	Split train/test 50% / 50% Repeat 10 000 times	Decision Tree		88% 82%	89% 90%		M1 MΦ M2 MΦ

HGG: High Grade Glioma; LDA=Linear discriminant analysis; RF=Random Forest; KNN=K-Nearest Neighbors; QDA=Quadratic Discriminant Analysis; SVM=Support Vector Machine; LogReg=Logistic Regression; CNN/NN=(Convolutional) Neural-Network; LOOCV/LOPOCV/CV=(Leave-One-(Patient)-Out) Cross-Validation; ACC=Accuracy; SE=Sensitivity; SP=Specificity; AUC=(Receiver operating characteristic) Area Under the Curve.

**Table 4.** Summary of the FLIm features and the data format used by the study examined in 8. For more information about the data, such as medical details, we refer the reader to the paper itself. The filter reference column describes the study cited to justify the channel filter/bandpass used, which can be visualised in figure 8.

Author	Filter reference	Lifetime	Features	data	Sample
Bianchetti *et al* [41]		Bi-Exponential	I_NAD(P)H_, *τ*_NAD(P)H_, I_FAD_, *τ*_FAD_	20 images 18-22 cells/images	512px×512px
Phipps *et al* [4]	Yankelevich *et al* [187]	Laguerre	*τ*_390_, *τ*_466_, *τ*_542_, *τ*_629_	20 tissue 14 patients	112,468px
Unger *et al* [5]		Laguerre	Per channel: *τ*_avg_, I_avg_, 12 Laguerre coefficients	18 patients & samples	323,026px after erosion
Marsden *et al* [109]		Laguerre	Decay for 1D-CNN. Per channel: *τ*_avg_, I_avg_, 12 Laguerre coefficients	50 *in vivo*, 53 *ex vivo* (20 tonsil, 23 tongue, 10 *other*)	76,695px *in vivo* 67,893px *ex vivo*
Marsden *et al* [10]		Laguerre	*τ*_390_, *τ*_470_, *τ*_542_	15 patients (9 lymph, 15 adipose, 15 thyroid, 15 parathyroid)	29,393 pixels 41 runs
Weyers *et al* [3]		Laguerre	Per channel: *τ*_avg_, I_ratios_, 12 Laguerre coefficients	55 patients train (38 tonsil, 17 tongue) 6 test (HNSCCUP)	2355 points sample (average)
Jo *et al* [136]	Cheng *et al* [188]	Bi-exponential	I_452_/I_500_ ratio. Per channel: *τ*_avg_, *τ*_1_, *τ*_2_, I, I_*n*_. For each feature: difference with the pixel value and image average. 6 features used only	73 patients (53 benign, 6 mDYS, 14 SCC)	
Duran *et al* [138]		Bi-exponential	Per channel: I, *τ*_avg_, (*τ*, *α*)_1_, *τ*_2_ 3 intensity ratios (I_*a*_/I_*b*_) 3 sum intensity ratios ((I_*a*_+I_*b*_)/I_*c*_)	68 train (34 healthy/cancer), 23 test	
Walsh *et al* [148]	Skala *et al* [43]	Bi-exponential	Cell size, Optical Redox Ratio, NAD(P)H (*τ*, *α*)_1_, *τ*_2_, *τ*_avg_ FAD (*τ*, *α*)_1_, *τ*_1_, *τ*_2_, *τ*_avg_	6 donors	8,355 cells
Qian *et al* [150]		Bi-exponential	Optical Redox Ratio, NAD(P)H (*τ*, *α*)_1_, (*τ*, *α*)_2_, *τ*_avg_, I FAD (*τ*, *α*)_1_, (*τ*, *α*)_2_, *τ*_avg_, I		11,218 cells (train) 19,245 cells (test)
Wang *et al* [119]		RLD	*τ*, I	≈70,000 images	128px×128px
Wang *et al* [104]		RLD	*τ*, I	24,554 images	128px×128px
Neto *et al* [152]	huang *et al* [189]	Bi-exponential	Optical redox Ratio, *τ*_1_, (*τ*, *α*)_2_, *τ*_avg_	6 donors	1,153 cells
Dunkers *et al* [149]	no filter	Bi-exponential	I, *τ*_1_, *τ*_2_, p_1_, (G, S) (Phasor)	19,182px/399 obj SP, 21,381px/205 obj HK, 3,324px/33 FP	43,887px 631 objects 3,324px/class when balanced
Cosci *et al* [139]		Bi-exponential	NAD(P)H (*τ*, *α*)_1_, (*τ*, *α*)_2_ FAD *τ*, *α*)_1_, (*τ*, *α*)_2_	101 measurement (55 healthy, 30 mDYS, 15 mrDYS	5px (measured point)
Butte *et al* [147]		Laguerre	*τ*_380, 440, 440, 550_, AVG(*τ*_440+450+460_)/AVG(*τ*_540+550_), AVG(*τ*_380+390_)/AVG(*τ*_440+450+460_), I_370_/I_380_, I_370_/I_390_, I_390_/I_440_, I_440_/I_370_, I_440_/I_380_, I_440_/I_450_, I_450_/I_370_, I_450_/I_550_, I_460_/I_370_, I_460_/I_390_, I_460_/I_450_, I_460_/I_540_, I_460_/I_550_, I_540_/I_440_, I_550_/I_380_, LEC-0_60_, LEC-0_450_, LEC-0_460_, LEC-0_550_, LEC-1_370_, LEC-1_390_, LEC-1_440_, LEC-1_450_, LEC-1_540_,	71 regions 35 normal cortex, 12 normal white matter, 7 low grade glioma, 5 anaplastic oligodendroglioma, 1 glioma with radiation necrosis	Single point
Romano *et al* [141]		Bi-exponential	Per channel: I, I_norm_, (*τ*, *α*)_1_, *τ*_2_, *τ*_avg_	76 samples 38 each class	140px×140px
Vasanthakumari *et al* [142]		Bi-exponential	Per channel: (*τ*, *α*)_1_, *τ*_2_, *τ*_avg_, I. I_390/452_, I_390/500_, I_452/500_. Per frequence: distance, spread, angle, symmetry	30 patients, 60 samples 41 benign, 19 malignant	140px×140px
Kröger *et al* [153]		Bi-exponential	(*τ*, *α*)_1_, (*τ*, *α*)_2_, *τ*_avg_, *τ*_2_/*τ*_1_, *α*_1_/*α*_2_, (*α*_1_-*α*_2_)/(*α*_1_+*α*_2_)	25 patients	399 cells
Ji *et al* [146]		Bi-exponential	*α*_2_, *τ*_avg_	71 patients 11 cancers, 7 CNI 18 Benign, 23 Normal 12 Follow-up	256 px × 256 px

## 4. Conclusion, challenges and opportunities

In the past few years, the use of FLIm applications in ML has grown exponentially. It appears that DL is on track to becoming a crucial component of lifetime estimation pipelines, thanks to the significant improvements it offers in both time savings and performance. Through successful proof of concept experiments, FLIm image classification and segmentation methods have demonstrated their effectiveness. As a result, we are now witnessing the publication of the first robust models, which further validate the potential of these methods [104, 137]. Many advancements which could benefit FLIm research still require attention. The main limiting factor is public data availability, which restricts both current FLIm research and the arrival of new ML researchers in the FLIm community. Datta *et al* [35] highlighted in their review the importance of dissemination to the community and standardisation of FLIm tools and methodology. We suggest two approaches for the current FLIm researchers to improve the quality of the ML research using FLIm data.

• Improvement and clarification of the methodology used, listing all parameters of interest in a clear and easy-to-read manner (tables 2–4 and figure 8 can be used as inspiration). Additionally, studies should include standardised metrics adapted to the problem as well as those included in similar papers, for comparison purposes.
• The creation of open-source datasets, extensively described and easily usable. A step further would be the creation of an ML contest featuring such a dataset to promote the field.

The benefits of the first step would be to aim for improved readability of publication, easier reproducibility, and the facilitation of easier comparison between studies. Whereas the creation of open source datasets could improve lifetime estimation, FLIm analysis as well as be used on both tasks at the same time, in a similar fashion as what Caughlin *et al* [137] did by training a joint autoencoder on the raw decay-curve and a classifier on the output of the encoder to classify the images created. The benefit of open-source datasets can be observed in other medical modalities. The creation of the ROSE dataset [192] and the OCTA-500 [193, 194] have pushed higher the number of DL-based OCTA vessel segmentation papers, with many studies published using only open source datasets [195–197]. Additionally, as mentioned, an ML contest could be created featuring such datasets. The Camelyon dataset (a breast cancer histopathology whole-slide images dataset) [198] challenge attracted a wide number of ML researchers, generating an important research output and providing a dataset for pre-training medical models. Kaggle^5^ 
^5^

kaggle.com/
 is a popular website for hosting datasets and competitions, which could also be used for hosting such a contest.

However, open-source datasets will not solve FLIm disparity between setups and experiments. A universal FLIm classifier does not appear to be possible, because of the difference between tissue from different organs, biological environments, fluorophores, exogenous and endogenous fluorescence and other factors leading to disparities in FLIm data. Applications so far have focused on singular tasks such as discriminating between two types of skin cancer. Standardising to some degree FLIm methodologies (on the spectral channel used or the fluorophores targeted, for example) would bring the distribution of the generated images closer, to which DL is sensitive. As highlighted by [58], lifetime can be affected by the experimental setup and other biological factors, including inter-patient variability. This creates challenges but also opportunities for the implementation of transfer learning, which has been proven efficient for cancer in other modalities [199–201] by pre-training DL models on similar medical data from different modalities or pathologies. As FLIm data are limited and not easy to obtain, being able to use lung FLIm images to enhance cervical cancer classification in FLIm images, for example, would be very helpful but may prove to be highly difficult, if possible at all, because of the different obstacles we cited. If so, the dissemination of publicly available datasets would be even more important to allow the use of DL. Ideally, public datasets should share similar configurations and parameters and be properly annotated, explained and detailed.

Nonetheless, to close the bridge in between the state-of-the-art in FLIm research and DL, we discuss here general improvements that can be applied to a majority of the studies discussed in this review. DL models are now readily available online in public repositories, or in multiple DL libraries such as PyTorch [202] or Tensorflow [203] and should be used in research. They can be trained from scratch, or used with pre-trained weights, usually obtained from Imagenet [204], and then fine-tuned. Such architectures or frameworks include ResNet [180] and its variants [205] for classification or regression, UNet [25] and other fully-convolutional NN for segmentation, and similarly UNet, or UNet-based frameworks such as VoxelMorph, as well as SuperPoint [206] or SuperGlue [207] for registration. Classifications models can be directly applied to replace ML models such as RF or SVM, while segmentation models such as UNet are better adapted for segmentation than pixel-by-pixel classification. The majority of models are trained on RGB images, and may need their input layer to be changed for FLIm images, which possesses different colour channels.

In more depth, multiple techniques can be implemented to make DL models better. Residual connections, introduced by ResNet, are now commonly used in the majority of DL models regardless of their applications. Classical convolutional block, along with convolutional layer, will be made with batch normalisation [208] or layer normalisation [209] and activation function such as Relu [210] or Gelu [211]. However, diverse improvements can be made to those blocks, such as squeeze-and-excitation block [212] or split-attention [213], both for channel attention to re-calibrate features and show channel features relationships. Other channels and spatial attention methods can be used, including convolutional block attention [214] or dual attention block [215]. As it can be observed, attention is greatly used to improve DL models, especially since the introduction of transformers [17], which led to the development of visual transformers [216], notably the Swin transformers [217]. Those mechanisms of self-attention should be considered when implementing DL models for FLIm, especially channel-wise attention, since FLIm possesses complicated inter-channel relationships. Attention mechanism would benefit FLIm for spatial dependency, spectral/channel dependency, as well as time-dependency. Another application to attention mechanism would be use of pair of images, such as intensity and lifetime, which could be mixed together in more advanced way than concatenating or weighting them together. In regards to state-of-the-art implementations of DL, ConvNeXts [218] present similar performance to the previously mentioned visual transformers model while using only pure CNNs, effectively updating the standard ResNet to today’s best practice. Additionally, transformers have displayed impressive results on time-series data, and could be applied to FLIm and lifetime data, in the same was as recurrent NN. We also want to highlight once more the research by [137] who used an autoencoder to generate features from the raw FLIm data, discarding the lifetime or phasor variables. This approach was possible because of the joint training with a subsequent task, here cancer classification, relying on the automatically generated features. This is akin to using classification models such as ResNet, which creates their own features representation before the final layer, and are often used this way in pre-training for feature extraction [219].

We suggest additional techniques and effort going through the training and to compensate for the lack of data in FLIm. Data augmentation can be used to increase the number of samples to train on, applying different modifications, from simple flipping or rotation for images, to more complicated approaches for different types of data [220, 221]. However, we do no suggest using the state-of-the-art data augmentation used in images classifications directly without adapting them speficially to FLIm. Examples such as Mixup [222] or Cutmix [223] may creates images largely out of the expected data distribution. Few-shot learning is used to train models on a small amount of data by comparing the sample tested with other existing samples of the same class [224]. It often takes advantage of transfer learning [225], mostly through pre-training on big datasets such as Imagenet. Transfer learning is useful to train a model to recognise high level features. For example, a model may learn to recognise the shape of cells while being trained on breast histology images, which it will be able to apply to FLIm images.

As we explained, additional modalities are used to provide ground truth and annotation, such as histopathology images. If FLIm usage were to increase in clinical settings, experts would need to be trained to interpret those images as they are trained to use the current gold standards. However, this lack of direct labelling makes it more difficult for humans to verify and trust the model result.

Complimentary image modalities can offer additional information. Fluorescence and lifetime signals are slowly varying functions of wavelength/wavenumber in comparison with Raman signals. The higher information content of Raman signals yields highly specific signatures of the molecules being probed, which opens up new opportunities for ML applications [58]. Raman signals, however, are generally much weaker than fluorescence, require higher laser powers or longer exposure times and can be obscured by fluorescence and noise. Histogramming TCSPC sensors enable the simultaneous acquisition of fluorescence and Raman signals [65], offering complementary spectroscopic analysis prospects[226]. Large and growing libraries of known Raman signals are also available for which signal similarity algorithms can be used to identify the molecule being probed [227, 228]. Together with FLIm, it could improve many medical and biomedical tasks. Additionally, time-correlated fluorescence technology could also be improved by mixing features other than the lifetime. ML algorithms may be able to pick up on hidden links between features that we do not see. For example, phasor plot variables [69] have been used together with lifetime by [149], showing better results than with phasor or lifetime features alone.

The future of FLIm imaging is intrinsically linked to the progress of both ML and hardware. As the technology advances the overall performance of FLIm devices will improve, making them more reliable and trustworthy medical technology. Performance improvements, such as speed and sensitivity, will not only enhance the quality of data collected but also aid the performance of ML algorithms applied to it. These advancements will significantly contribute to the development of *in vivo*, real-time, non-invasive FLIm imaging, which can be a game-changer in detecting various diseases, including cancer.

As ML algorithms become more sophisticated, they can help to improve the accuracy of FLIm data analysis and provide more precise diagnostic results. Additionally, hardware improvements can reduce image noise, allowing for better signal-to-noise ratios, and increasing the sensitivity of FLIm devices, which would be particularly useful in detecting early-stage diseases.

Ultimately, it is crucial to continue the investigation in both ML and hardware advancements to paving the way for the widespread use of FLIm technology in the future, transforming medical diagnosis and treatment, especially in the early detection of cancer and other diseases.

## Data Availability

No new data were created or analysed in this study.
